# Persistent Coughing as the First Symptom of Primary Mucinous Appendiceal Adenocarcinoma

**DOI:** 10.14740/jocmr2192w

**Published:** 2015-06-09

**Authors:** Stavros Gourgiotis, Christianna Oikonomou, Paraskevi Kollia, Evangelos Falidas, Constantinos Villias

**Affiliations:** a First Surgical Department, 417 NIMTS Military Veterans’ Fund Hospital of Athens, Greece

**Keywords:** Appendix, Mucinous, Adenocarcinoma, Diagnosis, Coughing

## Abstract

Primary appendiceal adenocarcinomas are extremely rare entities. Preoperative diagnosis is very difficult and is mainly based on computed tomography (CT) scan findings. Furthermore, in many cases, difficulties in establishing an accurate intraoperative diagnosis have resulted in a two-stage surgical intervention. We herein report a case of a primary appendiceal mucinous adenocarcinoma in a 67-year-old Caucasian man who presented with atypical symptoms of persistent coughing and weight loss. The chest CT showed lesions with features favorable of malignancy. Further investigation with abdominal CT and colonoscopy revealed a large tumor of the cecum expanding to the ascending colon. Typical right hemicolectomy was performed and the histopathological examination confirmed mucinous adenocarcinoma of the appendix. As some cases are accidentally discovered, the presented case describes an extremely rare first presentation of this tumor and emphasizes that the preoperative diagnosis of appendiceal cancer is challenging due to the lack of specific symptoms and signs.

## Introduction

Primary tumors of the appendix are unusual. Carcinoids are revealed in almost 85% of the cases whereas appendiceal adenocarcinomas are very rare malignant neoplasms with about 0.12 cases per 1,000,000 individuals diagnosed annually [1], accounting for 0.05-0.2% of all appendectomies and only 6% of all malignant tumors of the appendix [2]. They constitute < 0.5% of all gastrointestinal neoplasms [3]. Apart from their rarity, the spectrum of malignant disease is complex and has led to confusion concerning the accurate description of the natural history of these tumors. Appendiceal adenocarcinomas are classified into four groups: mucinous adenocarcinoma, colonic type adenocarcinoma, goblet cell carcinoma, and signet ring cell carcinoma [1]. Appendiceal carcinomas are usually well differentiated mucinous adenocarcinoma and do not show metastatic spread until late in the disease process. The diagnosis of primary appendiceal mucinous adenocarcinoma usually depends on the pathology following appendectomy or other explorative surgical procedures [4].

We herein report an extremely rare case of appendiceal adenocarcinoma in a 67-year-old male with first presentation of persistent coughing.

## Case Report

A 67-year-old Caucasian man presented with a persistent coughing for approximately 1 month. His symptom was not accompanied with loss of appetite, weight loss, or changes in bowel habits. The patient was smoking for about 25 years (approximately 40 pack-years) despite a 27-year abstinence from smoking. On laboratory investigation, peripheral blood counts and biochemical markers were within normal limits, except for a high rate of LDH. Serum concentrations of carcinoembryonic antigen (CEA), carbohydrate antigen (CA19-9), and alpha fetoprotein were within normal range as well.

**Figure 1:**
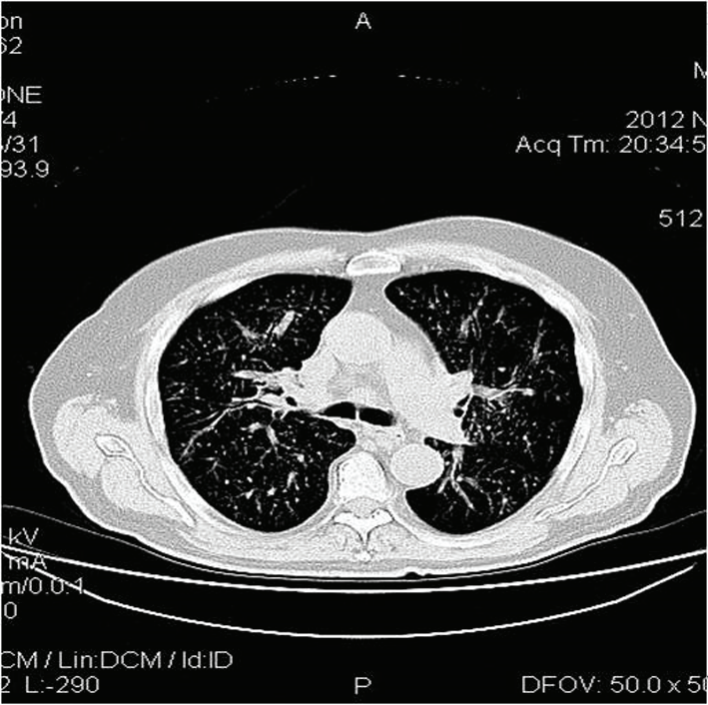
Chest CT shows multiple diffuse smooth thickening of the interstitial pulmonary parenchyma and an expanded right paratracheal lymph node with a diameter of 2.5 cm.

**Figure 2:**
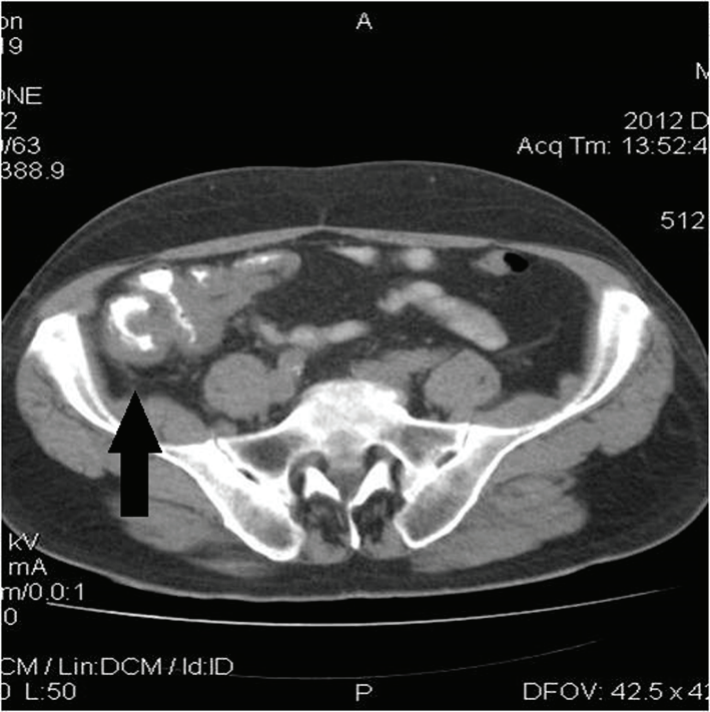
Enhanced abdominal CT demonstrates wall thickening of the cecum, the distal portion of the terminal ileum, and the lower portion of the ascending colon accompanied with pericolic inflammation, as indicated by the arrow.

Chest computed tomography (CT) showed multiple diffuse smooth thickening of the interstitial pulmonary parenchyma as well as an enlargement of the right paratracheal lymph nodes with a maximum diameter of 2.5 cm (Fig. 1). Contrast-enhanced abdominal CT scans revealed wall thickening of the cecum, the distal portion of the terminal ileum and the lower portion of the anion colon accompanied with pericolic inflammation and enlargement of the paracolic lymph nodes of the region (Fig. 2). No hepatic metastases were recognized. Main paraortic lymph nodes were observed with a maximum diameter of 2 cm.

**Figure 3:**
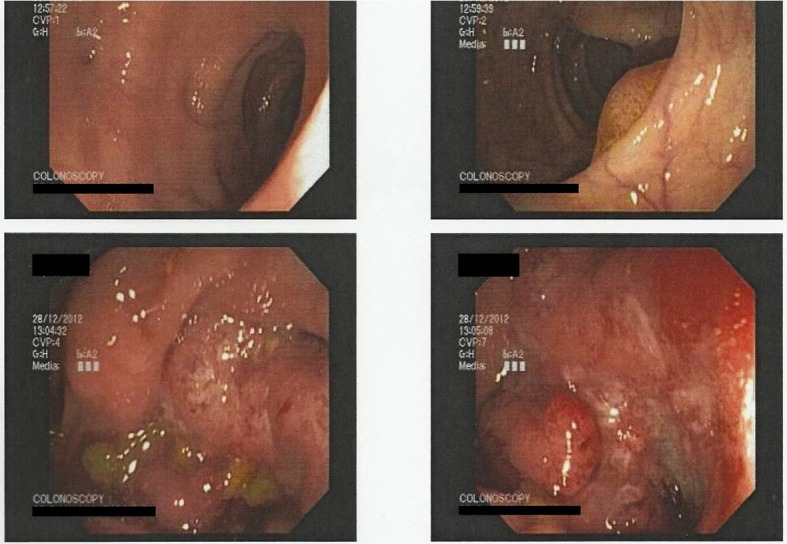
Colonoscopy reveals the large mass of the ascending colon.

**Figure 4:**
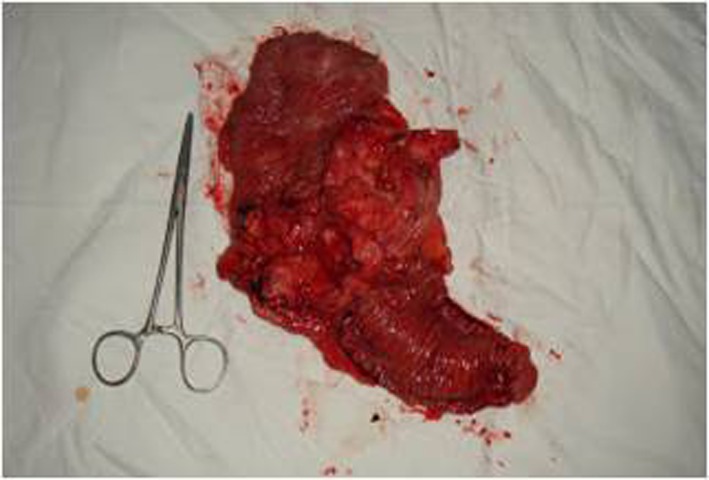
The resected specimen of right hemicolectomy.

A colonoscopy was performed revealing a large mass in the cecum expanding in the lower portion of the ascending colon (Fig. 3). Biopsies were taken showing adenocarcinoma. Furthermore, the cytological examination of the cells from the bronchoalveolar lavage (BAL) revealed glandular tumor cells. The patient, following oncological council, subjected to neoadjuvant chemoradiation, in accordance with the therapeutic protocol for metastatic adenocarcinoma of colon with non-excludable pulmonary metastasis. Six months later, a right hemicolectomy (Fig. 4) with side-to-side ileocolic anastomosis was performed. Has also be mentioned that no peritoneal metastases were observed in the presented case, although the patient had pulmonary metastases.

**Figure 5:**
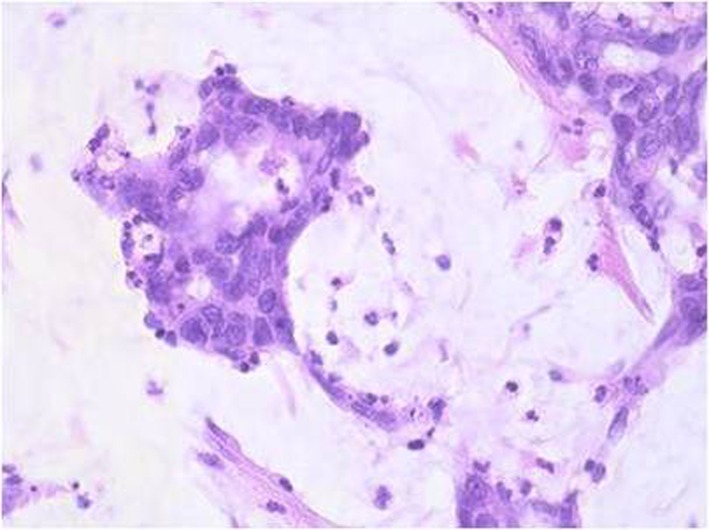
Dilated carcinomatous structures with extracellular production of mucus (H&E stain, original magnification, × 25).

Histopathological examination of the resected specimen revealed a poor differentiated appendiceal mucinous adenocarcinoma (Fig. 5) whereas a negative staining for CD56, chromogranin, and synaptophysin was noted. Venous and lymphatic (all the 35 resected lymph nodes) invasion was observed whereas surgical margins were free of tumor cells. The patient’s postoperative course was complicated by evisceration which was managed by surgical procedure and he was discharged 15 days after the initial operation. The patient received adjuvant chemotherapy and died 1 year after surgery.

## Discussion

There are four major histological subtypes of appendiceal adenocarcinoma: cystic, colonic, carcinoid, and adenocarcinoid. Carcinoids are the most common, comprising nearly 90% of all primary appendiceal tumors while mucinous cystadenocarcinoma is the second most common type. Primary appendiceal cancer is diagnosed in 0.9-1.4% of appendectomy specimens [5].

Clinical presentation of appendiceal tumors usually is non-specific and misleading, thus accurate preoperative diagnosis is difficult and errors in the selected treatment can occur. Some are entirely asymptomatic. They are never suspected preoperatively and seldom intraoperatively and the diagnosis is usually made at histopathological examination of the surgical specimen or as an incidental finding during exploration for another disease [1]. Adenocarcinoma of appendix most often presents as acute appendicitis or as a palpable abdominal mass [6]. The mean age of presentation is 50 years old with a prevalence of the male gender 4:1 [7]. In other cases, this entity can mimic an ovarian tumor especially when there is intraabdominal spread or pseudomyxoma peritonei [8]. In our case, the patient had no specific gastrointestinal symptoms, thus unless imaging results, it was difficult to suspect the disease; the only mentioned symptom was the persistent coughing.

Intestinal endoscopy, barium enema, and selective ileocolic arteriography have been used for the preoperative diagnosis but no established method currently exists. In the same pattern, the role of colonoscopy is controversial. According to a previous review, appendiceal lesions were recognized during colonoscopy in only 11% of the patients with appendiceal adenocarcinoma [9]. Differently, CT imaging offers 95% sensitivity in revealing lesions of the vermiform appendix either as abnormalities in the shape or as an increase in dimension, and thus it is an essential tool in the differential diagnosis [10].

Treatment of appendiceal adenocarcinoma depends on the stage of the disease. When limited in the appendix, right hemicolectomy is the treatment of choice, whereas the presence of distant metastases or peritoneal spread requires aggressive debulking followed with neoadjuvant and adjuvant chemoradiotherapy along with it [11]. However, in case of pulmonary metastasis the treatment consists of neoadjuvant chemoradiation, in accordance with the therapeutic protocol for metastatic adenocarcinoma of colon, and right hemicolectomy later. The prognosis of this tumor depends on the grade of malignancy and the success of debulking surgery to remove all the tumors that have metastasized into the abdomen. Although distant metastasis and visceral involvement of appendiceal mucinous adenocarcinoma are very rare, our patient had both of them: pulmonary metastases with venous and lymphatic (all the 35 resected lymph nodes) invasion. However, no peritoneal metastases were found during the surgical procedure.

The purpose of this report was to highlight that a suspicion of appendiceal carcinoma is fundamental, when pathology is focalized near the ileocolic valve so as to eliminate errors concerning the therapeutic strategy. In the present case, the patient’s clinical presentation was confusing and led us to initially ignore the possibility of appendiceal carcinoma. The signs and symptoms of an appendiceal adenocarcinoma were not specifically apparent, but the CT and the colonoscopy were useful to identify a correlation with the colon. However, the final diagnosis was established by the histopathological examination of the resected specimen.

## References

[1] McCusker ME, Cote TR, Clegg LX, Sobin LH (2002). Primary malignant neoplasms of the appendix: a population-based study from the surveillance, epidemiology and end-results program, 1973-1998. Cancer.

[2] Alexiou K, Sikalias N, Demonakou M, Mylona SC, Triantafyllis V, Kalogirou A, Antsaklis G (2009). Mucinous adenocarcinoma of the appendix presenting with atypical symptomatology and presence of pseudomyxoma peritonei: a case report. Cases J.

[3] Rutledge RH, Alexander JW (1992). Primary appendiceal malignancies: rare but important. Surgery.

[4] Ito H, Osteen RT, Bleday R, Zinner MJ, Ashley SW, Whang EE (2004). Appendiceal adenocarcinoma: long-term outcomes after surgical therapy. Dis Colon Rectum.

[5] Connor SJ, Hanna GB, Frizelle FA (1998). Appendiceal tumors: retrospective clinicopathologic analysis of appendiceal tumors from 7,970 appendectomies. Dis Colon Rectum.

[6] Behera PK, Rath PK, Panda R, Satpathi S, Behera R (2011). Primary appendiceal mucinous adenocarcinoma. Indian J Surg.

[7] Conte CC, Petrelli NJ, Stulc J, Herrera L, Mittelman A (1988). Adenocarcinoma of the appendix. Surg Gynecol Obstet.

[8] Yoshimura M, Terai Y, Konishi H, Tanaka Y, Tanaka T, Sasaki H, Ohmichi M (2013). Laparoscopic diagnosis of adenocarcinoma of the appendix mimicking serous papillary adenocarcinoma of the peritoneum. Case Rep Obstet Gynecol.

[9] Trivedi AN, Levine EA, Mishra G (2009). Adenocarcinoma of the appendix is rarely detected by colonoscopy. J Gastrointest Surg.

[10] Pickhardt PJ, Levy AD, Rohrmann CA, Kende AI (2002). Primary neoplasms of the appendix manifesting as acute appendicitis: CT findings with pathologic comparison. Radiology.

[11] Gonzalez-Moreno S, Sugarbaker PH (2004). Right hemicolectomy does not confer a survival advantage in patients with mucinous carcinoma of the appendix and peritoneal seeding. Br J Surg.

